# Association of Extracellular Membrane Vesicles with Cutaneous Wound Healing

**DOI:** 10.3390/ijms18050956

**Published:** 2017-05-01

**Authors:** Uyen Thi Trang Than, Dominic Guanzon, David Leavesley, Tony Parker

**Affiliations:** 1 Tissue Repair and Translational Physiology Program, Institute of Health and Biomedical Innovation, Queensland University of Technology, Kelvin Grove, Queensland 4059, Australia; thitranguyen.than@hdr.qut.edu.au (U.T.T.T.); dominic.guanzon@hdr.qut.edu.au (D.G.); 2 School of Biomedical Science, Faculty of Health, Queensland University of Technology, Kelvin Grove, Queensland 4059, Australia; 3 Wound Management Innovation Cooperative Research Centre, 25 Donkin, West End, Queensland 4101, Australia; d.leavesley@imb.a-star.edu.sg; 4 Institute of Medical Biology—Agency for Science, Technology and Research, 8A Biomedical Grove, Singapore 138648, Singapore

**Keywords:** wound healing, proliferation, migration, angiogenesis, extracellular membrane vesicles, microvesicles, apoptotic bodies, exosomes, endothelial cells, keratinocytes

## Abstract

Extracellular vesicles (EVs) are membrane-enclosed vesicles that are released into the extracellular environment by various cell types, which can be classified as apoptotic bodies, microvesicles and exosomes. EVs have been shown to carry DNA, small RNAs, proteins and membrane lipids which are derived from the parental cells. Recently, several studies have demonstrated that EVs can regulate many biological processes, such as cancer progression, the immune response, cell proliferation, cell migration and blood vessel tube formation. This regulation is achieved through the release and transport of EVs and the transfer of their parental cell-derived molecular cargo to recipient cells. This thereby influences various physiological and sometimes pathological functions within the target cells. While intensive investigation of EVs has focused on pathological processes, the involvement of EVs in normal wound healing is less clear; however, recent preliminarily investigations have produced some initial insights. This review will provide an overview of EVs and discuss the current literature regarding the role of EVs in wound healing, especially, their influence on coagulation, cell proliferation, migration, angiogenesis, collagen production and extracellular matrix remodelling.

## 1. Introduction

Membrane-enclosed extracellular vesicles (EVs) include apoptotic bodies, microvesicles and exosomes which are released by various cell types, and have been found in cell culture media, as well as body fluids such as breast milk, urine, amniotic fluids, saliva and blood [1,2,3,4,5,6,7,8,9,10,11]. During EV biogenesis, a number of biological molecules are encapsulated into the EV, including DNA, small RNAs, proteins and lipids which are derived from parental cells [6,11]. However, the real significance of EVs lies in their ability to deliver their contents to recipient cells, thereby altering biological and cellular processes. Furthermore, aberrant delivery of EV cargo to recipient cells has been implicated in several pathologies such as some autoimmune disorders and cancers [12,13,14,15].

**Figure 1:**
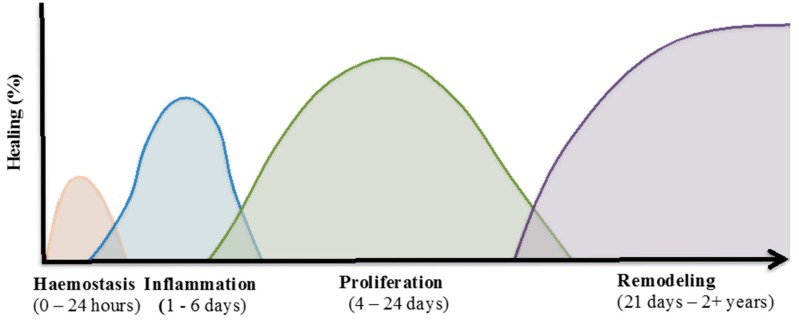
Wound healing process. The normal tissue repair process is comprised of continuous and overlapping phases. These four phases include: (i) Haemostasis; (ii) inflammation; (iii) proliferation; and (iv) remodelling. Each phase consists of different cellular events which requires the interplay of multiple cell populations [22].

Wound healing is a complicated process which involves an overlapping cascade of events characterised into four distinct phases (Figure 1). These include:

- Haemostasis, where blood loss ceases;
- The inflammatory phase, characterised by infiltration of immune cells to combat infection and remove cellular debris;
- The proliferative phase, where fibroblasts and keratinocytes at the wound margins migrate into the wound and increase in cell number to re-establish the barrier function of the skin; and
- The remodelling phase, during which reorganisation of the dermis occurs and the preliminary extracellular matrix (ECM), laid down during the earlier phases of the healing response, is remodelled to strengthen the wound area through the reduction of scar tissue [16].

Preliminary studies have shown that EVs may be involved in wound healing through the control of a number of cellular processes [17,18,19,20,21]. Therefore, this review will summarise the role of EVs derived from predominant cells involved in the wound healing process. An overview of EV classification, biogenesis, components, and cell-to-cell communication via EVs is also described.

## 2. Extracellular Membrane Vesicles (EVs)

EVs have a phospholipid bilayer similar to the cell membrane, with diameters ranging from 40 nm to 5 µm. In general, EVs can be classified into three subtypes: apoptotic bodies (also known as apoptotic vesicles); microvesicles (also known as shedding vesicles); and exosomes.

### 2.1. Apoptotic Bodies

Apoptotic bodies are the largest vesicle population, with a diameter ranging from 1 to 5 µm and have a heterogeneous morphology. Apoptotic bodies are released when cells undergo apoptosis and therefore they contain various components from their parental cells often including organelles and DNA fragments [23].

**Figure 2:**
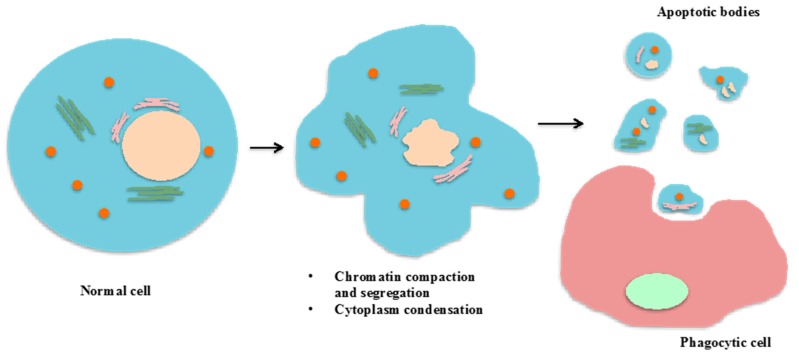
Formation of apoptotic bodies and clearance by phagocytosis. Formation of apoptotic bodies includes the condensation and segregation of the nucleus, and the deterioration and blebbing of the plasma membrane. The result of these processes is a separation of the cellular contents into membrane-enclosed vesicles which can be cleared by phagocytic cells.

Apoptosis and necrosis are major mechanisms of cell death, which produce cell debris but are activated by diverse biological stimuli [23,24,25]. In contrast to apoptosis which is programed cell death, necrosis is passive and activated by mechanical damage or disease [25]. Interestingly, apoptotic bodies are released into the extracellular environment through several stages. During the early and intermediate stages, the cell membrane is contracted, the cytoplasm is condensed, and the cells become smaller in size [23]. Simultaneously, nuclear chromatin is also condensed and undergoes alteration, and the plasma membrane deteriorates such that its permeability increases in the late stage. As a result, the plasma membrane undergoes a process that is commonly known as blebbing [24]; and the cellular content is disintegrated into distinct membrane enclosed vesicles known as apoptotic bodies [24]. Therefore, apoptotic bodies contain the cytoplasm, but does not necessarily include tightly packed organelles or nuclear fragments. However, if organelles are encapsulated within apoptotic bodies, these organelles have been shown to have their integrity maintained [23]. During the formation process of apoptotic bodies, phosphatidylserine (PS) residues that are normally located on the internal surface of the plasma membrane, subsequently translocate to the external surface. This process presents extracellular signals that attract macrophages to clear the apoptotic bodies via phagocytosis (Figure 2).

### 2.2. Microvesicles

**Figure 3:**
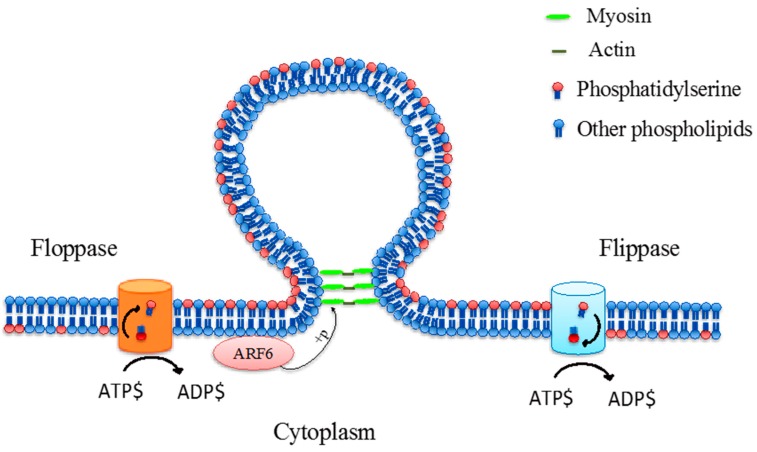
Phospholipid translocase activity via floppase and flippase which translocates phosphatidylserine and other phospholipids from the inner leaflet to the outer leaflet, and outer leaflet to inner leaflet, respectively, during microvesicle formation. These processes are adenosine triphosphate (ATP)-dependant [28,30,32,33].

Microvesicles, also known as “ectosomes”, are the second largest vesicle type between 100 and 1000 nm in diameter, which are formed by the outward budding and fission of the plasma membrane [26,27]. However, there is limited understanding about the formation and shedding mechanism of microvesicles at the cell surface, although it is hypothesised that the formation of membrane microvesicles may be a result of the dynamic interplay between phospholipid redistribution and cytoskeletal protein contraction [28,29]. This process is regulated by several enzymes such as calpain, flippase, floppase, scramblase and gelsolin [30]. Flippases transfer phospholipids from the outer leaflet to the inner leaflet while floppases transfer phospholipids from the inner leaflet to the outer leaflet [31]. The translocation of PS to the outer-membrane leaflet is a signal that induces the membrane budding/vesicle formation (Figure 3) [28]. In addition, microvesicle formation was associated with ADP-ribosylation factor 6 (ARF6), a small GTPase protein, which regulating the activation of myosin light chain kinase (MLCK) and subsequent phosphorylation of MLCK lead to a promotion of contraction of actin-based cytoskeleton [32]. Thereby, the budding process is completed through the contraction of cytoskeletal structures via actin and myosin interactions [32,33].

### 2.3. Exosomes

**Figure 4:**
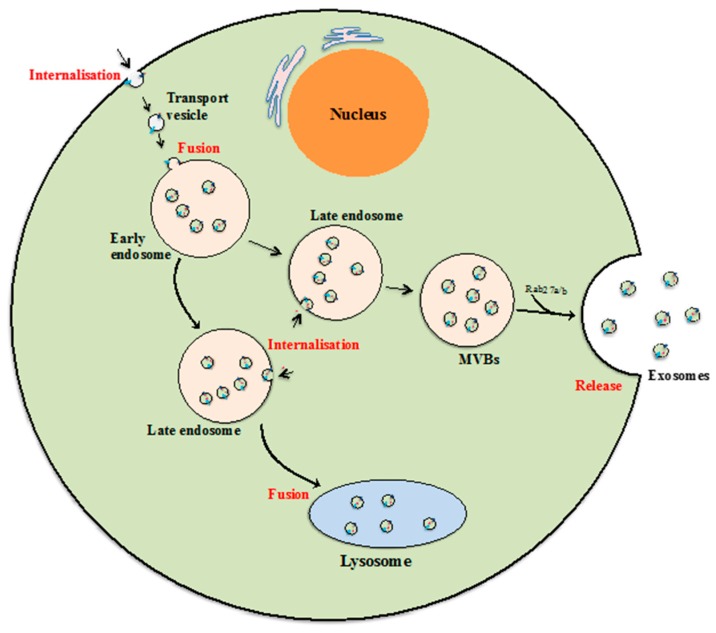
Exosome biogenesis. Beginning from internalization of membrane proteins and lipid complexes by endocytosis, endocytotic vesicles are delivered to early endosomes, which fuse with each other resulting in formation of late endosomes/multivesicular bodies (MVB). MVBs either release exosomes by fusion with the cellular membrane, or their contents are degraded if they fuse with lysosomes [40,42,43]. The key steps of the exosomal formation and development process are highlighted in red.

Exosomes are the smallest class of EVs with diameters between 40 and 100 nm and a cup shape morphology according to previous studies using electron microscopy [34]. Although the mechanism of exosome biogenesis is not fully understood, it is commonly accepted that exosomes are formed and developed via the endocytic pathway, and are subsequently released to the extracellular environment by exocytosis [35,36]. The formation and release process begins when fluids, solutes, macromolecules, plasma components and particles are internalized by various endocytic trafficking pathways into transport vesicles [37,38]. The transport vesicles then fuse with one another or with an existing sorting endosome to form early endosomes. During the next developmental stage, early endosomes may collect proteins and other components and develop into late endosomes, and that late endosome may recycle its components back to the plasma membrane or be subjected to degradation by lysosomes [37,39]. Alternatively, that late endosomes may develop to become multi-vesicular bodies (MVBs) carrying and releasing exosomes when MVBs fuse with the cellular membrane (Figure 4) [39,40]. Thus while complex and much remains unknown about exosome biogenesis, it is clear that their formation and release are tightly regulated by multiple signalling mechanisms. For instance, some evidence suggests that Endosomal Sorting Complexes Required for Transport (ESCRT) pathway is needed for exosome biogenesis [39,41] as is Rab27a/b which is involved in endosome development and docking of MVBs to the cellular plasma membrane [42].

## 3. Cell-to-Cell Communication

Cell-to-cell communication is a pivotal mechanism that enables the differentiation of cells and the development of multicellular organisms. The mechanisms of cell-to-cell communication are very complex and involve intercellular and intracellular signals, which includes cell junctions, adhesion contacts and soluble factors [44,45]. Cells can form bridges (cytonemes) to connect and exchange surface-associated cargo to neighbouring cells based on the mechanism of adhesion [46], or tunnelling nanotubes to contact and transfer both cell surface molecules and cytoplasmic components to other cells [46,47]. Recently, EVs were found to be a new means of communication because they carry functional molecules and can horizontally transfer these to neighbouring cells [19].

**Figure 5:**
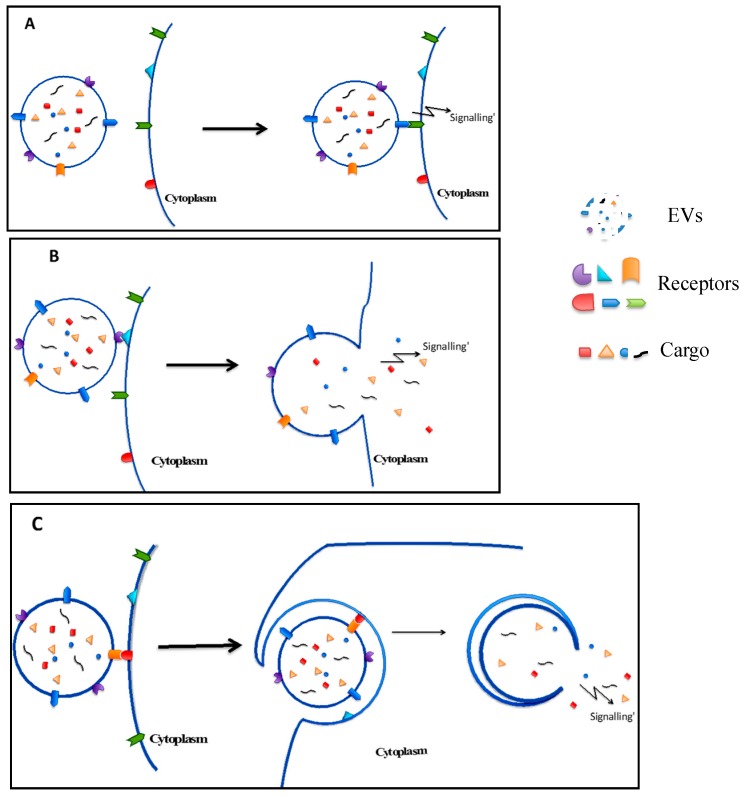
Interaction of EVs with target cells: (**A**) intracellular signalling due to EV membrane ligand cell surface receptor interactions [53,54]; (**B**) direct membrane fusion which induces cell function through release of EV cargo into target cells [55,56,57,58]; and (**C**) internalisation of EVs into target cells, prior to the release of their cargo into the recipient cell cytoplasm inducing functional effects [21,59].

Cell-to-cell communication by EVs is described as a way for cells to interact with neighbouring cells or one another over long distances, when EVs were detected in the circulation and other body fluids [44]. The binding of EVs to target cells is specific with examples including: platelet-derived EVs binding to neutrophils [48]; neutrophil-derived EVs binding to dendritic cells [49] or to monocytes and endothelial cells [50]; and leukocyte-derived EVs binding to platelets [51]. The interaction between EVs and target cells are thought to require the coordinated action of the cytoskeleton and vesicle fusion machinery [28]. Despite the limited understanding about EVs transport, different mechanisms of interaction between EVs and recipient cells include ligand–receptor interaction, internalisation and direct membrane fusion have been studied [52] (Figure 5).

Examples of ligand–receptor interaction mechanisms include studies of EVs released from dendritic cells and platelets [53,54]. Dendritic cell-derived exosomes contain MHC class II and CD9 on their membrane and were found to bind to the surface of activated T cells [53]. This binding could be an interaction between MHC II molecules on exosome membranes and T cell receptors on T cell membranes [53]. Interestingly, exosomes only bound to the surface of the plasma membrane without fusing and internalisation into T cells [53]. Similarly, platelet-derived microvesicles containing the CD41 antigen were discovered to bind to the membrane of human bone marrow CD34^+^ cells, which then stimulated the adhesion of these cells to the endothelium as well as directed them from peripheral blood back into the bone marrow [54].

Regarding the mechanism of direct membrane fusion, some evidences suggested that microvesicles fused directly with the plasma membrane and transferred their contents to the intracellular milieu of the recipient cells [55,56,57,58]. In the case of microvesicles enriched selectively with P-selectin glycoprotein ligand-1 (PSGL-1), the fusion may be controlled by Annexin V or antibody to PSGL-1 [55]. Additionally, it seems that the fusion of EVs to the plasma membrane is also dependant on SNARE proteins, which regulate the fusion and target specificity in intracellular vesicle trafficking [58]. When EVs fuse with and transfer membrane components such as receptors and ligands to their targets, these can increase the resistance to apoptosis in the case of macrophages receiving chemokine receptors; or induce an increased frequency of apoptosis in the case of T lymphocytes receiving the Fas ligand (a death-receptor ligand) [56,57].

Morelli et al. found evidence of the internalisation of circulating EVs into dendritic cells, phagocytes of spleen, and Kupffer cells in the liver via clathrin-dependent endocytosis [59]. This internalisation of EVs by dendritic cells requires participation of the dendritic cell cytoskeleton as well as surface molecules such as externalised PS, CD11a, CD54, CD9 and CD81 [59]. Additionally, cellular maturity also influences the internalisation of EVs by dendritic cells, since immature dendritic cells exhibit a higher internalisation capability than mature dendritic cells [59]. This internalisation of EVs into target cells could induce peripheral T-cell tolerance in the absence of danger signals [59] or stimulate cell proliferation and migration [21].

## 4. Functional Components of EVs

### 4.1. Proteins

In general, all three populations of mammalian EVs share some common characteristics such as structure (lipid bi-layer), and tend to have common types of cargo such as protein, lipid or genetic material. However, these characteristics may be distinct depending on the manner of formation as well as the nature of their parental cells. Specific molecules that are unique to each vesicle population can be used to distinguish between them. For example, PS is only transferred to the external plasma membrane surface when cells undergo apoptosis and where microvesicles are formed and shed [28,60]. In another example, the tetraspanin family (e.g., CD9, CD63, and CD81) form a complex network of interacting molecules which play a role in trafficking of transmembrane proteins [61]. Thus CD9, CD63, and CD81 are enriched and often detected in exosomes since they are often involved in exosome biogenesis pathways [8,52,62], however these components are also detected in other vesicles [63]. Interestingly, CD24 could be considered as a marker of exosomes isolated from urine and amniotic fluids [8]. Additionally, the CD24 has been found to be over-expressed in various cancer cell lines such as ovarian, breast, non-small cell lung, prostate and pancreatic carcinomas [64]. In terms of other functional proteins, Dujarin et al. reported that Tau, a microtubule-associated protein, and matrix metalloproteinase 1 (MMP-1) protein, were enriched in microvesicles compared to exosomes [17,65]. However, no studies have reported whether or not Tau and MMP-1 proteins are enriched in apoptotic bodies.

Importantly, specific EV biomolecular cargo has previously been linked to cancer and tumour development [50,66,67]. For example, apoptotic bodies, which contained DNA encoding the oncogenes H-rasV12 and c-myc, were found to induce tumour formation and growth in severe combined immunodeficient (SCID) mice [66]. Interestingly, the authors also found that these H-rasV12 and c-myc genes were replicated and incorporated into the recipient genome in newly formed tumour cells in vivo (SCID mice model) [66]. Furthermore, microvesicles carrying MMP-9 released from breast carcinoma cells, fibrosarcoma cells and polymorphonuclear leucocytes functioned in the digestion of the extracellular matrix, which is necessary for the progress of cancer growth and inflammation [50,67].

EVs have also been found to carry components associated with angiogenesis and coagulation processes [55,68,69]. For instance, endothelial cell-derived microvesicles, which were enriched with MMPs, could stimulate the invasion and formation of capillary-like structures of human umbilical vein endothelial cells (HUVECs) [69]. Indeed, MMPs have been identified as important mediators of angiogenesis [70,71], but exactly how the EV MMPs contribute to the angiogenic process has not yet been revealed. Interestingly, the accumulation of MMPs within microvesicles was shown to be stimulated by angiogenic factors including, fibroblast growth factor (FGF)-2 and vascular endothelial growth factor (VEGF) [69]. Moreover, EVs were also associated with coagulation since microvesicles contained tissue factor (TF) derived from platelets and monocytes [55,68].

EVs also have a function in the regulation of immune responses, since exosomes have been found to contain major histocompatibility complex (MHC) class I and II molecules and may transfer these molecules to dendritic cells [5,72,73]. The transferral of MHC I molecules from melanoma cell line-derived exosomes to dendritic cells, triggered the production of Interferon γ in these cells [73]. Furthermore, MHC I molecules carried by dendritic cell derived exosomes could activate CD8+ T cell responses [5,73]. These studies indicate that exosomes can potentially act as extracellular antigen sources, which could help to develop immune interventions.

Finally, cytoplasmic proteins, such as tubulin, actin, actin-binding proteins, annexins and Rab proteins, which are involved in intracellular membrane fusion and transport, and ESCRT machinery complex proteins are also found in exosomes [39,41,74,75]. In addition, molecules responsible for signal transduction, such as protein kinases, 14-3-3 and heterotrimeric G proteins, are enveloped during exosome formation and release [39,74,76,77]. While there is evidence to support the conclusion that the molecular components of EVs may be delivered to and elicit functional responses in recipient cells, unequivocal evidence is still required [19]. Taken together, these studies indicate that EVs and their cargo are functional and could influence recipient cell behaviour.

### 4.2. Lipids

Interestingly, EV lipid composition, which includes cholesterol, sphingomyelin, ceramide, phospholipids and glucans can provide an additional means of identification for exosomes [78,79,80,81]. Indeed, exosomes have been found to be enriched with higher amounts of cholesterol and sphingolipids, including sphingomyelin and hexosylceramide, but contain lower amounts of accumulated phosphatidylcholine, compared to parent cell membranes [81]. Importantly, Trajkovic et al. showed that high concentrations of ceramide exist in the micro-domains where multi-vesicular endosomes are formed. This led to the hypothesis that ceramide could be useful for enlarging micro-domains and facilitating the inducement of domain budding [81]. These studies indicate that lipids can partially regulate the formation and release of vesicles [78,81].

### 4.3. Genetic Materials (Messenger RNAs and miRNAs)

Regarding genetic biomolecules, RNAs including messenger RNAs (mRNAs) and microRNAs (miRNAs) have also been found in EVs which have been increasingly found to elicit various functions in recipient cells [11,82,83,84,85,86,87,88,89,90]. In particular, large amounts of mRNAs and miRNAs were found in all three EV types released from both cell cultures and body fluids [14,82,83,84,87,88,89,90]. Importantly, these EV-derived genetic molecules were selectively sorted into EVs and as such could be used for diagnostics, such as cancer markers, or as potential treatment targets [90,91,92,93,94]. In addition to carrying the information from secreting cells, EV mRNAs and miRNAs can also function to promote biological processes, including proliferation, angiogenesis and apoptosis [86,92,95,96]. Interestingly, there is evidence showing that EV mRNAs were horizontally transferred to recipient cells and are able to be translated into proteins [3,95,96]. Additionally, the proteins translated from transferred EV mRNAs have been shown to activate PI3K/AKT signalling pathways in recipient endothelial cells [96] and accelerate morphological and functional recovery in rat liver [95]. While these studies provide some evidence for the role of EV mRNAs and miRNAs in the regulation of biological processes, it is clear that further studies are required to more fully understand the functional consequences of EV genetic cargo.

## 5. The Role of EVs in Cutaneous Wound Healing

Wound healing is an essential process that enables the restoration of the structure and function of damaged or injured tissues. For most tissues, the process requires multiple cell types from several distinct lineages where each responds to and generates a range of signals at different times [97]. The wound healing process involves a series of overlapping phases including: haemostasis; inflammation; migration; and tissue remodelling [16]. During these phases, a series of precise biological events occur including: rapid vascular spasms, platelet plug formation and coagulation (collectively known as haemostasis); inflammation; cell migration into the wound site; cell proliferation and differentiation to form granulation tissue; angiogenesis; and extracellular-matrix reorganisation [97]. Evidence for the involvement of EVs in wound healing has been described for coagulation, cell proliferation, cell migration and angiogenesis and summarised in Table 1 [17,18,19,21].

### 5.1. Coagulation

**Figure 6:**
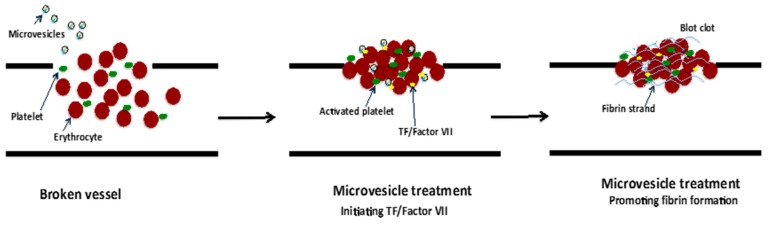
Promotion of coagulation by TF-barring microvesicle treatment. Rapid coagulation is triggered by the initiating TF/Factor VII and promotion of fibrin strand formation [98,99].

The blood coagulation cascade is complex and regulated by a range of factors such as calcium ions, PS and TF in addition to many others. TF is an initiator of coagulation activation that was found to be present on the plasma membrane of EVs, including microvesicles and exosomes, which were derived from various cell types such as human monocytes/macrophages [55] human platelets [98]; and EVs isolated from human saliva [99]. Indeed, monocyte-derived microvesicles enriched with TF and PSDL-1 were found to bind, fuse and transfer their content to collagen-activated platelets [55]. The result of this TF transfer from microvesicles to the platelets enabled TF to become “decrypted”/activated, and thus initiate the extrinsic coagulation cascade leading to conversion of prothrombin to thrombin and fibrin clot formation [55]. Furthermore, in an in vitro study, salivary EVs that contain TF in association with coagulation factor VII significantly reduced the clotting time of human wound blood (Figure 6) [99]. Similarly, EVs carrying TF from pericardial blood of cardiac surgery patients induced coagulation in vitro and stimulated thrombogenicity in rats [98]. Conversely, EVs from the venous blood of healthy individuals also carrying TF did not reduce clotting time [98]. This may be due to soluble inhibitors including Tissue Factor Pathway Inhibitor (TFPI) or conformational regulation of TF in plasma that impedes the binding of coagulation factor VII and inhibits its activity [55].

### 5.2. Cell Proliferation

Cell proliferation, a pivotal cellular process underpinning wound healing, has been shown to be regulated by EVs derived from a plethora of cell types, such as human mesenchymal stem cells (MSCs) [21,100,101], fibroblasts [18], murine embryonic stem cells [102], and human endothelial progenitor cells [103]. The regulation of cell proliferation by EVs occurred through the binding to or internalisation of EVs into recipient cells and delivery of EV content such as RNAs [104,105]. For instance, the internalisation of EVs into fibroblasts greatly enhanced the cell proliferation due to the activation of the Akt and Erk1/2 effector pathway by EVs [18,21,100,106,107]. Additionally, embryonic stem cell-derived EVs may facilitate fibroblast proliferation, possibly through the activation of the mitogen-activated protein kinase (MAPK) pathway [102]. Importantly, these aforementioned activated pathways induce the expression of a number of genes involved in cell cycle progression such as c-myc, cyclin A1 and cyclin D2 which then support cell proliferation [21,106]. Furthermore, activation of these pathways also promoted the production of growth factors, including insulin-like growth factor-1 (IGF-1), stromal-derived growth factor-1 (SDF-1) and cytokine interleukin-6 (IL-6), VEGF-α and transforming growth factor beta (TGF-β) [18,21,101,102]. Thus, it seems clear that EVs can promote cell proliferation by activation of signalling pathways not only directly involved in stimulation of cell cycle but also involved in the regulation of growth factor expression. Additionally, this growth factor expression can act in a paracrine or autocrine fashion to further stimulate cell growth responses.

### 5.3. Migration

Following clotting, immune cells including neutrophils and macrophages are recruited to remove necrotic tissues, debris, and bacteria from the wound site. Within hours after injury, other cells including epithelial cells and fibroblasts migrate into the wound site to perform specific tasks, such as induction of growth factor secretion, the synthesis of extracellular matrix, angiogenesis and stimulation of wound closure [97]. Preliminary studies have been conducted investigating the involvement of EVs in the migration of various cells associated with cutaneous repair, such as human and murine epithelial cells [108], bovine endothelial cells [109], human MSCs [100], human keratinocytes and fibroblasts in vitro [110].

Evidently, keratinocyte migration has been shown to be influenced by EVs released from various cell types; however, different results were observed depending on the specific biomolecules carried by the EVs [110,111]. For example, CoCl_2_-treated tumour cell-derived exosomes which contained C4.4A (Ly6/PLAUR domain-containing protein 3), α6β4 integrin and MMP-14 inhibited keratinocyte migration through degradation of laminin 332 resulting in delayed wound closure [111]. Conversely, keratinocyte derived exosomes with heat-shock protein (HSP) 90α cargo significantly enhanced the migration of primary human keratinocytes in a wound scratch model [110]. Additionally, the migration of endothelial cells, which are crucial for vascular repair and regeneration, has also been shown to be influenced by EVs released from cells such as human keratinocytes [108], human MSCs [112,113]; and bovine endothelial cells [109]. However, the mechanisms that facilitate EV mediated endothelial migration are unclear.

In a wound healing context, fibroblasts are known to release growth factors which induce other cells to proliferate and migrate, and also produce collagen (COL) that provides structure to the wound [16,114]. Interestingly, EVs have been shown to regulate the migration of fibroblasts towards wound sites [104,105,110]. Cheng et al. showed that TGFα stimulates HSP90α secretion from human keratinocytes via the exosome pathway [110]. Most exosomes contain some member of the heat-shock protein (HSP) family, such as HSP70 and HSP90 [39,76,115]. This protein family supports the folding of nascent peptides; prevents the aggregation of proteins; assists with the transportation of other proteins across cell membranes [116], and can induce cell motility [115,117]. Interestingly, human keratinocyte conditioned media containing exosomes with HSP90α cargo was found to stimulate the rapid migration of dermal fibroblasts [110]. HSP90α is comprised of four domains, including an N-terminal domain, a charged sequence connected to the N-terminal domain, a middle domain and a C-terminal domain [118]. It is thought that HSP90α may promote cell migration through binding interactions with the cell surface receptor LRP-1/CD91 [110,117]. Furthermore, in corroboration with cell migration enhancement, EVs released from mesenchymal stem cells and keratinocytes were also shown to promote the expression of important genes such as TGF-β, transforming growth factor beta receptor II, COL I, COL III, N-cadherin, cyclin-1, MMP-1, MMP-3 and IL-6 in fibroblasts [104,105]. These genes are involved in the Erk1/2 signalling pathway, which has roles in both cell proliferation and migration.

### 5.4. Angiogenesis

New blood vessel formation is critical for wound healing in order to supply nutrients and oxygen to newly formed tissues. The formation of new blood vessels requires the proliferation of endothelial cells, as well as the interaction between endothelial cells, angiogenic factors (such as VEGF and fibroblast growth factor) and surrounding ECM proteins [119]. Under chemotaxis, endothelial cells penetrate the underlying vascular basement membrane, invade ECM stroma and form tube-like structures that continue to extend, branch, and create networks [119,120]. Interestingly, all three populations of EVs (apoptotic bodies, microvesicles and exosomes) contribute to the regulation of vessel formation by enhancing the expression of pro-angiogenic factors [21,92,102,103,106,107,109,112,120,121]. For example, exosomes released from human embryonic MSCs [92,113] and human endothelial cells [103,106] enhanced angiogenesis by promoting endothelial cell proliferation and migration towards the wound site. Furthermore, a large number of blood vessels were observed in exosome-treated sites compared to control treatment [106,107,112].

**Figure 7:**
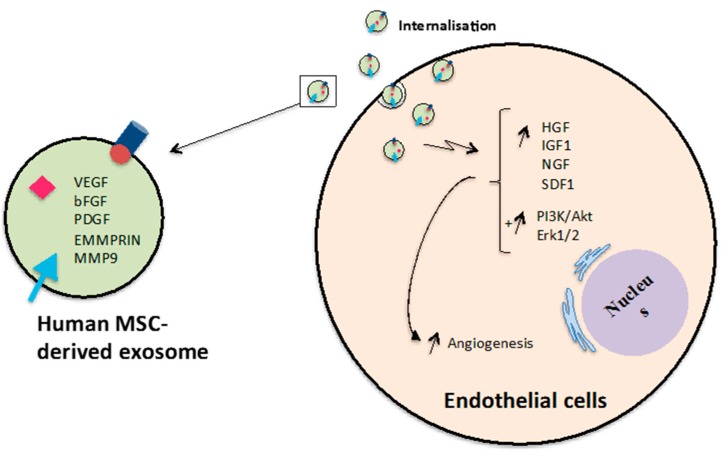
Promotion of angiogenesis by MSC-derived exosomes. Exosomes released from human MSCs can induce expression of genes and activate PI3K/Akt and Erk1/2 signalling pathways in endothelial cells leading to promotion tube formation and newly formed vessels.

Interestingly, various critical pro-angiogenic genes, including IL-6, IL-8, angiopoietin-1, E-selectin, and fibroblast growth factor 2, which activate the Erk1/2 signalling pathway, were up-regulated after platelet-rich plasma and endothelial progenitor cell-derived exosome stimulation [106,122]. Additionally, the downstream target of the Erk1/2 signalling pathway such as inhibitor of DNA binding 1, cyclooxygenase 1, VEGFA, VEGFR-2, c-Myc and cyclin-D1, were also increased after treatment with exosomes [106,122,123]. This provides further evidence that exosomes enhance endothelial cell function through activation of the Erk1/2 signalling pathway (Figure 7) [106]. In contrast, microvesicles from multiple cellular sources inhibited tube-like structure formation of microvascular endothelial cells via CD36 on the treated cell membrane, while exosome derived from the same cell source as the microvesicles did not have the same inhibitory activity [124]. Taken together, the previous studies imply that EVs could potentially be used in vascular regenerative medicine. Moreover, the EV bioactivities may depend on EV types and the physiological conditions of administered cells However, further research is required to investigate which specific EV derived biomolecules are responsible for the stimulation of angiogenesis.

### 5.5. Collagen Production and ECM Remodelling

Successful wound healing requires the contribution of many cellular events and biological processes in addition to coagulation, cell proliferation, cell migration and angiogenesis. Besides the involvement of EVs in the above events, EVs have also been shown to regulate ECM remodelling which is the last phase of wound healing. For example, EVs have been shown to stimulate an increase in elastin secretion, which is a structural protein of the ECM [102,112]. Furthermore, when wound sites are treated with MSC-derived exosomes, an increase of COL I and III were observed in the early stage of wound healing [105,112,125]. However, in the late stage of wound healing, exosomes may inhibit collagen expression [105]. Interestingly, EVs catalyse the crosslinking of collagen in the ECM via lysyl oxidase-like 2 (LOXL2), which is located on the exterior of the exosome membrane [126]. In addition, a study by Huleihel et al. (2016) observed that EVs present within the ECM were closely associated with the collagen network of the matrix, but these EVs could be separated from the fibre network [127]. This may indicate that EVs attend to the ECM formation and function. Furthermore, EVs have also been demonstrated to significantly increase the healing rate and reduce scar widths by interaction with Annexin A1 and formyl peptide receptors in a rat model [107,108,125]. Taken together, these studies indicate that EVs play a pivotal role in the ECM remodelling phase of wound healing.

## 6. Conclusions

In the past, EVs were disregarded simply as cellular debris. However, current research has demonstrated that EVs contain bioactive molecules and are able to deliver these to recipient cells via newly described cell-to-cell communication mechanisms. In wound healing, EVs were initially shown to regulate inflammation, proliferation, migration, angiogenesis, collagen production and ECM remodelling. Such regulation could be mediated through the enhancement of gene expression, suppression of gene translation, and/or activation of signalling pathways important for wound healing processes. However, the current level of knowledge regarding how the EV molecular cargo regulates the process of wound healing remains unclear. Therefore, more research is required to clarify the regulation of EVs during the wound healing process, and to translate these results to the clinic.

## Figures and Tables

**Table 1:** Summary of research investigating EVs involvement in wound healing.

Events	EV Types	Parental Cells	Target Cells	Secreted Factors/Factors Presented in EVs	Molecules/Pathways Activated	References
Coagulation	Mv, Ex	Saliva/granulocytes, EPC	Blood	TF	Trigger coagulation by initiating TF/factor VII	[99]
	Mv	Monocytes	Activated Platelet	TF, PSGL-1		[55]
	Mv	Plasma/Platelet, erythrocytes, granulocytes	Blood	TF	Promote thrombus formation	[98]
Proliferation	Ex	MSC	FB, EC		Increase expression levels of HGF, IGF1, NGF, SDF1; increase re-epithelialisation; reduce scar widths; promote collagen maturity and the creation of newly formed vessels; accelerate maturation of wound sites; activate Akt, Erk and Stat3 signalling	[21,112]
	Ex/nanoparticles *	ESC	FB		Enhance the expression levels of mRNA, EVGFα, TGFβ, collagen I, Ki-67	[102]
	Mv, Ex	MyoFB	FB, EC			[18]
	Ex	KC	FB	HSP90	Could not promote cell proliferation	[110]
	Ex	Platelet-rich plasma	EC	VEGF, bFGF, PDGFBB	Activating Pi3K/Akt and Erk signalling pathway	[107]
	Ex	EC, EPC	EC		Activating Erk1/2 signalling pathway	[103,106]
	Mv	Platelet-rich plasma after exercise	HUVEC			[128]
Migration	Ex	MSC	FB, EC		Induction the expression of HGF, IGF1, NGF, SDF1; activate Akt, Erk and Stat3 signalling	[21,112]
	Ex	KC	KC, HDMECs	HSP90	Hsp90-Ex increased cell migration without the need to bind any cofactor or ATP; CD91 is receptor of extracellular Hsp90; TGFβ could not inhibit cell migration	[110]
	Ex/Ev	EC	Murine wound	Annexin-I		[108]
	Ex/nanop-articles *	ESC	FB		Higher expression of mRNA, EVGFα, TGFβ, collagen I, Ki-67	[102]
	Ex	Platelet-rich plasma	EC	VEGF, bFGF, PDGFBB	Activating Pi3K/Akt and Erk signalling pathway	[107]
	Ex	EC, EPC	EC		Activating Erk1/2 signalling pathway	[103,106]
	Ex	hUSC	HUVEC			[121]
	Ev	Multiple cellular sources	EC	CD63		[124]
Angiogenesis	Ex	MSC	FB, EC		Induction the expression of HGF, IGF1, NGF, SDF1; promote the creation and maturation of newly formed vessels, increase re-epithelialisation, reduce scar widths	[21,112]
	Ex/nanop-articles*	ESC	FB		Enhance the expression levels of mRNA, EVGFα, TGFβ, collagen I, Ki-67	[102]
	Mv, Ex	MyoFB	Fb, EC	VEGF, FGF2	Increase angiogenesis	[18]
	Ex	Platelet-rich plasma	EC	VEGF, bFGF, PDGFBB	Activating Pi3K/Akt and Erk signalling pathway	[107]
	Ex	EC	EC		Activating Erk1/2 signalling pathway	[103,106]
	Ex	MSC	EC	EMMPRIN, VEGF, MMP9	ERK/Akt pathway	[123]
	Ex	MSC	EC	miR-125a	Direct target DLL4	[92]
	Ex	hUSC	HUVEC			[121]
	Ex	Epithelium cells	EC	VEGFR		[129]
	Ev	Multiple cellular sources	EC	CD63		[124]
	Mv	Platelet-rich plasma after exercise	HUVEC			[128]
Collagen production and ECM remodelling	Ex	MSC	FB	TF	Increase reepithelialisation, reduce scar widths, promote collagen maturity and maturation of wound sites	[112]
	Ex	MSCs, FB	FB		Increase collagen production	[105,125]
	Ex	Hypoxic EC	ECM	LOXL2	ECM remodelling	[126]

FB: Fibroblasts, KC: Keratinocytes, MSCs: Mesenchymal stem cells, EC: Endothelial cells, ESC: Embryonic stem cells, EPC: Epithelial cells, hUSC: Human urine derived stem cells, HUVUEC: Human umbilical vein endothelial cells, Mv: Microvesicle(s), Ex: Exosome(s), EMMPRIN: Extracellular matrix metalloproteinase inducer, (*) nanoparticles mimicking. Exosome were extracted from living cells.

## Abbreviations

ARF6	ADP-ribosylation factor 6
COL	Collagen
DNA	Deoxyribonucleic acid
EC(s)	Endothelial cell(s)
ECM	Extracellular matrix
EMMPRIN	Extracellular matrix metalloproteinase inducer
EPC(s)	Epithelial cell(s)
ESC(s)	Embryonic stem cell(s)
ESCRT	Endosomal sorting complex required for transport
EV(s)	Extracellular membrane vesicle(s)/extracellular vesicle(s)
Ex	Exosome(s)
FB	Fibroblast
FGF	Fibroblast growth factor
HSP	Heat-shock protein
HUVEC(s)	Human umbilical vein endothelial cell(s)
hUSC	human urine derived stem cells
IGF	Insulin-like growth factor
IL	Interleukin
KC(s)	Keratinocyte(s)
LOXL2	Lysyl oxidase-like 2
MAPK	Mitogen-activated protein kinase
MHC	Major histocompatibility complex
miRNA(s)	microRNAs(s)
MLCK	Myosin light chain kinase
MMP	Matrix metalloproteinase
MSC(s)	Mesenchymal stem cell(s)
Mv	Microvesicle(s)
MVB(s)	Multi-vesicular bodie(s)
P	Platelet
PS	Phosphatidylserine
PDGFBB	Platelet-derived growth factor-BB
PSGL-1	P-selectin glycoprotein ligand-1
RNA	Ribonucleic acid
SCID	Severe combined immunodeficient
SDF	Stroma-derived growth factor
TF	Tissue factor
TFPI	Tissue factor pathway inhibitor
TGF	Transforming growth factor
VEGF	Vascular endothelial growth factor

## References

[1] Baietti M.F., Zhang Z., Mortier E., Melchior A., Degeest G., Geeraerts A., Ivarsson Y., Depoortere F., Coomans C., Vermeiren E. (2012). Syndecan-syntenin-ALIX regulates the biogenesis of exosomes. Nat. Cell Biol..

[2] Meister G., Landthaler M., Peters L., Chen P.Y., Urlaub H., Lührmann R., Tuschl T. (2005). Identification of novel argonaute-associated proteins. Curr. Biol..

[3] Valadi H., Ekström K., Bossios A., Sjöstrand M., Lee J.J., Lötvall J.O. (2007). Exosome-mediated transfer of mRNAs and microRNAs is a novel mechanism of genetic exchange between cells. Nat. Cell Biol..

[4] Vlassov A.V., Magdaleno S., Setterquist R., Conrad R. (2012). Exosomes: Current knowledge of their composition, biological functions, and diagnostic and therapeutic potentials. Biochim. Biophys. Acta.

[5] Hao S., Bai O., Yuan J., Qureshi M., Xiang J. (2006). Dendritic cell-derived exosomes stimulate stronger CD8^+^ CTL responses and antitumor immunity than tumor cell-derived exosomes. Cell. Mol. Immunol..

[6] Lee T.H., D’Asti E., Magnus N., Al-Nedawi K., Meehan B., Rak J. (2011). Microvesicles as mediators of intercellular communication in cancer—The emerging science of cellular “debris”. Semin Immunopathol..

[7] Michael A., Bajracharya S.D., Yuen P.S., Zhou H., Star R.A., Illei G.G., Alevizos I. (2010). Exosomes from human saliva as a source of microRNA biomarkers. Oral Dis..

[8] Keller S., Rupp C., Stoeck A., Runz S., Fogel M., Lugert S., Hager H.-D., Abdel-Bakky M., Gutwein P., Altevogt P. (2007). CD24 is a marker of exosomes secreted into urine and amniotic fluid. Kidney Int..

[9] Zonneveld M.I., Brisson A.R., van Herwijnen M.J., Tan S., van de Lest C.H., Redegeld F.A., Garssen J., Wauben M.H., Nolte-‘t Hoen E.N. (2014). Recovery of extracellular vesicles from human breast milk is influenced by sample collection and vesicle isolation procedures. J. Extracell. Vesicles.

[10] Rupp A.-K., Rupp C., Keller S., Brase J.C., Ehehalt R., Fogel M., Moldenhauer G., Marmé F., Sültmann H., Altevogt P. (2011). Loss of epcam expression in breast cancer derived serum exosomes: Role of proteolytic cleavage. Gynecol. Oncol..

[11] Crescitelli R., Lässer C., Szabo T.G., Kittel A., Eldh M., Dianzani I., Buzás E.I., Lötvall J. (2013). Distinct RNA profiles in subpopulations of extracellular vesicles: Apoptotic bodies, microvesicles and exosomes. J. Extracell. Vesicles.

[12] Skriner K., Adolph K., Jungblut P.R., Burmester G.R. (2006). Association of citrullinated proteins with synovial exosomes. Arthritis Rheum..

[13] Ostenfeld M.S., Jeppesen D.K., Laurberg J.R., Boysen A.T., Bramsen J.B., Primdal-Bengtson B., Hendrix A., Lamy P., Dagnaes-Hansen F., Rasmussen M.H. (2014). Cellular disposal of miR23b by RAB27-dependent exosome release is linked to acquisition of metastatic properties. Cancer Res..

[14] Mittelbrunn M., Gutiérrez-Vázquez C., Villarroya-Beltri C., González S., Sánchez-Cabo F., González M.Á., Bernad A., Sánchez-Madrid F. (2011). Unidirectional transfer of microRNA-loaded exosomes from T cells to antigen-presenting cells. Nat. Commun..

[15] Yáñez-Mó M., Siljander P.R.-M., Andreu Z., Bedina Zavec A., Borràs F.E., Buzas E.I., Buzas K., Casal E., Cappello F., Carvalho J. (2015). Biological properties of extracellular vesicles and their physiological functions. J. Extracell. Vesicles.

[16] Guo S.A., di Pietro L.A. (2010). Factors affecting wound healing. J. Dent. Res..

[17] Keerthikumar S., Gangoda L., Liem M., Fonseka P., Atukorala I., Ozcitti C., Mechler A., Adda C.G., Ang C.-S., Mathivanan S. (2015). Proteogenomic analysis reveals exosomes are more oncogenic than ectosomes. Oncotarget.

[18] Moulin V.J., Mayrand D., Messier H., Martinez M.C., Lopez-Vallé C.A., Genest H. (2010). Shedding of microparticles by myofibroblasts as mediator of cellular cross-talk during normal wound healing. J. Cell. Physiol..

[19] Cocucci E., Racchetti G., Meldolesi J. (2009). Shedding microvesicles: Artefacts no more. Trends Cell Biol..

[20] Rani S., Ritter T. (2016). The exosome—A naturally secreted nanoparticle and its application to wound healing. Adv. Mater..

[21] Shabbir A., Cox A., Rodriguez-Menocal L., Salgado M., Badiavas E.V. (2015). Mesenchymal stem cell exosomes induce proliferation and migration of normal and chronic wound fibroblasts, and enhance angiogenesis in vitro. Stem Cells Dev..

[22] Velnar T., Bailey T., Smrkolj V. (2009). The wound healing process: An overview of the cellular and molecular mechanisms. J. Int. Med. Res..

[23] Elmore S. (2007). Apoptosis: A review of programmed cell death. Toxicol. Pathol..

[24] Kerr J.F., Wyllie A.H., Currie A.R. (1972). Apoptosis: A basic biological phenomenon with wide-ranging implications in tissue kinetics. Br. J. Cancer.

[25] Edinger A.L., Thompson C.B. (2004). Death by design: Apoptosis, necrosis and autophagy. Curr. Opin. Cell Biol..

[26] Simpson R., Mathivanan S. (2012). Extracellular microvesicles: The need for internationally tecognised nomenclature and stringent purification criteria. J. Proteom. Bioinform..

[27] Kalra H., Simpson R.J., Ji H., Aikawa E., Altevogt P., Askenase P., Bond V.C., Borràs F.E., Breakefield X., Budnik V. (2012). Vesiclepedia: A compendium for extracellular vesicles with continuous community annotation. PLoS Biol..

[28] Akers J.C., Gonda D., Kim R., Carter B.S., Chen C.C. (2013). Biogenesis of extracellular vesicles (EV): Exosomes, microvesicles, retrovirus-like vesicles, and apoptotic bodies. J. Neurooncol..

[29] Maezawa S., Yoshimura T., Hong K., Duzgunes N., Papahadjopoulos D. (1989). Mechanism of protein-induced membrane fusion: Fusion of phospholipid vesicles by clathrin associated with its membrane binding and conformational change. Biochemistry.

[30] Piccin A., Murphy W.G., Smith O.P. (2007). Circulating microparticles: Pathophysiology and clinical implications. Blood Rev..

[31] Devaux P.F., Herrmann A., Ohlwein N., Kozlov M.M. (2008). How lipid flippases can modulate membrane structure. Biochim. Biophys. Acta.

[32] Muralidharan-Chari V., Clancy J., Plou C., Romao M., Chavrier P., Raposo G., D’Souza-Schorey C. (2009). ARF6-regulated shedding of tumor cell-derived plasma membrane microvesicles. Curr. Biol..

[33] McConnell R.E., Higginbotham J.N., Shifrin D.A., Tabb D.L., Coffey R.J., Tyska M.J. (2009). The enterocyte microvillus is a vesicle-generating organelle. J. Cell Biol..

[34] Théry C., Ostrowski M., Segura E. (2009). Membrane vesicles as conveyors of immune responses. Nat. Rev. Immunol..

[35] Raposo G., Nijman H.W., Stoorvogel W., Liejendekker R., Harding C.V., Melief C.J., Geuze H.J. (1996). B lymphocytes secrete antigen-presenting vesicles. J. Exp. Med..

[36] Pan B.-T., Teng K., Wu C., Adam M., Johnstone R.M. (1985). Electron microscopic evidence for externalization of the transferrin receptor in vesicular form in sheep reticulocytes. J. Cell Biol..

[37] Harrison P., Gardiner C., Sargent I.L. (2014). Extracellular vesicles in health and disease.

[38] Huotari J., Helenius A. (2011). Endosome maturation. EMBO J..

[39] Théry C., Zitvogel L., Amigorena S. (2002). Exosomes: Composition, biogenesis and function. Nat. Rev. Immunol..

[40] Harding C., Heuser J., Stahl P. (1983). Receptor-mediated endocytosis of transferrin and recycling of the transferrin receptor in rat reticulocytes. J. Cell Biol..

[41] Wang Z., Hill S., Luther J.M., Hachey D.L., Schey K.L. (2012). Proteomic analysis of urine exosomes by multidimensional protein identification technology (MudPIT). Proteomics.

[42] Ostrowski M., Carmo N.B., Krumeich S., Fanget I., Raposo G., Savina A., Moita C.F., Schauer K., Hume A.N., Freitas R.P. (2010). RAB27a and RAB27b control different steps of the exosome secretion pathway. Nat. Cell Biol..

[43] Raposo G., Stoorvogel W. (2013). Extracellular vesicles: Exosomes, microvesicles, and friends. J. Cell Biol..

[44] Camussi G., Deregibus M.C., Bruno S., Cantaluppi V., Biancone L. (2010). Exosomes/microvesicles as a mechanism of cell-to-cell communication. Kidney Int..

[45] Majka M., Janowska-Wieczorek A., Ratajczak J., Ehrenman K., Pietrzkowski Z., Kowalska M.A., Gewirtz A.M., Emerson S.G., Ratajczak M.Z. (2001). Numerous growth factors, cytokines, and chemokines are secreted by human CD34^+^ cells, myeloblasts, erythroblasts, and megakaryoblasts and regulate normal hematopoiesis in an autocrine/paracrine manner. Blood.

[46] Sherer N.M., Mothes W. (2008). Cytonemes and tunneling nanotubules in cell-cell communication and viral pathogenesis. Trends Cell Biol..

[47] Rustom A., Saffrich R., Markovic I., Walther P., Gerdes H.-H. (2004). Nanotubular highways for intercellular organelle transport. Science.

[48] Lösche W., Scholz T., Temmler U., Oberle V., Claus R.A. (2004). Platelet-derived microvesicles transfer tissue factor to monocytes but not to neutrophils. Platelets.

[49] Eken C., Gasser O., Zenhaeusern G., Oehri I., Hess C., Schifferli J.A. (2008). Polymorphonuclear neutrophil-derived ectosomes interfere with the maturation of monocyte-derived dendritic cells. J. Immunol..

[50] Gasser O., Hess C., Miot S., Deon C., Sanchez J.-C. (2003). Characterisation and properties of ectosomes released by human polymorphonuclear neutrophils. Exp. Cell. Res..

[51] Pluskota E., Woody N.M., Szpak D., Ballantyne C.M., Soloviev D.A., Simon D.I., Plow E.F. (2008). Expression, activation, and function of integrin αMβ2 (Mac-1) on neutrophil-derived microparticles. Blood.

[52] Smith J., Leonardi T., Huang B., Iraci N., Vega B., Pluchino S. (2015). Extracellular vesicles and their synthetic analogues in aging and age-associated brain diseases. Biogerontology.

[53] Nolte E.N., Buschow S.I., Anderton S.M., Stoorvogel W., Wauben M.H. (2009). Activated T cells recruit exosomes secreted by dendritic cells via LFA-1. Blood.

[54] Janowska-Wieczorek A., Majka M., Kijowski J., Baj-Krzyworzeka M., Reca R., Turner A.R., Ratajczak J., Emerson S.G., Kowalska M.A., Ratajczak M.Z. (2001). Platelet-derived microparticles bind to hematopoietic stem/progenitor cells and enhance their engraftment. Blood.

[55] Del Conde I., Shrimpton C.N., Thiagarajan P., López J.A. (2005). Tissue-factor–bearing microvesicles arise from lipid rafts and fuse with activated platelets to initiate coagulation. Blood.

[56] Kim J.W., Wieckowski E., Taylor D.D., Reichert T.E., Watkins S., Whiteside T.L. (2005). Fas ligand–positive membranous vesicles isolated from sera of patients with oral cancer induce apoptosis of activated T lymphocytes. Clin. Cancer Res..

[57] Mack M., Kleinschmidt A., Brühl H., Klier C., Nelson P.J., Cihak J., Plachý J., Stangassinger M., Erfle V., Schlöndorff D. (2000). Transfer of the chemokine receptor CCR5 between cells by membrane-derived microparticles: A mechanism for cellular human immunodeficiency virus 1 infection. Nat. Med..

[58] Fader C.M., Sánchez D.G., Mestre M.B., Colombo M.I. (2009). Ti-VAMP/VAMP7 and VAMP3/cellubrevin: Two v-SNARE proteins involved in specific steps of the autophagy/multivesicular body pathways. Biochim. Biophys. Acta.

[59] Morelli A.E., Larregina A.T., Shufesky W.J., Sullivan M.L., Stolz D.B., Papworth G.D., Zahorchak A.F., Logar A.J., Wang Z., Watkins S.C. (2004). Endocytosis, intracellular sorting, and processing of exosomes by dendritic cells. Blood.

[60] Bilyy R.O., Shkandina T., Tomin A., Muñoz L.E., Franz S., Antonyuk V., Kit Y.Y., Zirngibl M., Fürnrohr B.G., Janko C. (2012). Macrophages discriminate glycosylation patterns of apoptotic cell-derived microparticles. J. Biol. Chem..

[61] Berditchevski F., Odintsova E. (2007). Tetraspanins as regulators of protein trafficking. Traffic.

[62] Escola J.M., Kleijmeer M.J., Stoorvogel W., Griffith J.M., Yoshie O., Geuze H.J. (1998). Selective enrichment of tetraspan proteins on the internal vesicles of multivesicular endosomes and on exosomes secreted by human B-lymphocytes. J. Biol. Chem..

[63] Kowal J., Arras G., Colombo M., Jouve M., Morath J.P., Primdal-Bengtson B., Dingli F., Loew D., Tkach M., Théry C. (2016). Proteomic comparison defines novel markers to characterize heterogeneous populations of extracellular vesicle subtypes. Proc. Natl. Acad. Sci. USA.

[64] Kristiansen G., Machado E., Bretz N., Rupp C., Winzer K.-J., König A.-K., Moldenhauer G., Marmé F., Costa J., Altevogt P. (2010). Molecular and clinical dissection of CD24 antibody specificity by a comprehensive comparative analysis. Lab. Investig..

[65] Dujardin S., Bégard S., Caillierez R., Lachaud C., Delattre L., Carrier S., Loyens A., Galas M.-C., Bousset L., Melki R. (2014). Ectosomes: A new mechanism for non-exosomal secretion of tau protein. PLoS ONE.

[66] Bergsmedh A., Szeles A., Henriksson M., Bratt A., Folkman M.J., Spetz A.-L., Holmgren L. (2001). Horizontal transfer of oncogenes by uptake of apoptotic bodies. Proc. Natl. Acad. Sci. USA.

[67] Dolo V., Ginestra A., Cassarà D., Violini S., Lucania G., Torrisi M.R., Nagase H., Canevari S., Pavan A., Vittorelli M.L. (1998). Selective localization of matrix metalloproteinase 9, β1 integrins, and human lymphocyte antigen class I molecules on membrane vesicles shed by 8701-BC breast carcinoma cells. Cancer Res..

[68] Heijnen H.F., Schiel A.E., Fijnheer R., Geuze H.J., Sixma J.J. (1999). Activated platelets release two types of membrane vesicles: Microvesicles by surface shedding and exosomes derived from exocytosis of multivesicular bodies and α-granules. Blood.

[69] Taraboletti G., D’Ascenzo S., Borsotti P., Giavazzi R., Pavan A., Dolo V. (2002). Shedding of the matrix metalloproteinases MMP-2, MMP-9, and MT1-MMP as membrane vesicle-associated components by endothelial cells. Am. J. Pathol..

[70] Giavazzi R., Taraboletti G. (2001). Preclinical development of metalloproteasis inhibitors in cancer therapy. Crit. Rev. Oncol. Hematol..

[71] Hidalgo M., Eckhardt S.G. (2001). Development of matrix metalloproteinase inhibitors in cancer therapy. J. Natl. Cancer Inst..

[72] Blanchard N., Lankar D., Faure F., Regnault A., Dumont C., Raposo G., Hivroz C. (2002). TCR activation of human T cells induces the production of exosomes bearing the TCR/CD3/ζ complex. J. Immunol..

[73] Wolfers J., Lozier A., Raposo G., Regnault A., Théry C., Masurier C., Flament C., Pouzieux S., Faure F., Tursz T. (2001). Tumor-derived exosomes are a source of shared tumor rejection antigens for CTL cross-priming. Nat. Med..

[74] Théry C., Boussac M., Véron P., Ricciardi-Castagnoli P., Raposo G., Garin J., Amigorena S. (2001). Proteomic analysis of dendritic cell-derived exosomes: A secreted subcellular compartment distinct from apoptotic vesicles. J. Immunol..

[75] Théry C., Regnault A., Garin J., Wolfers J., Zitvogel L., Ricciardi-Castagnoli P., Raposo G., Amigorena S. (1999). Molecular characterization of dendritic cell-derived exosomes. J. Cell Biol..

[76] Bard M.P., Hegmans J.P., Hemmes A., Luider T.M., Willemsen R., Severijnen L.-A.A., van Meerbeeck J.P., Burgers S.A., Hoogsteden H.C., Lambrecht B.N. (2004). Proteomic analysis of exosomes isolated from human malignant pleural effusions. Am. J. Respir. Cell Mol. Biol..

[77] Wang T., Feng Y., Sun H., Zhang L., Hao L., Shi C., Wang J., Li R., Ran X., Su Y. (2012). miR-21 regulates skin wound healing by targeting multiple aspects of the healing process. Am. J. Pathol..

[78] Batista B.S., Eng W.S., Pilobello K.T., Hendricks-Muñoz K.D., Mahal L.K. (2011). Identification of a conserved glycan signature for microvesicles. J. Proteome Res..

[79] Laulagnier K., Motta C., Hamdi S., Sébastien R., Fauvelle F., Pageaux J.-F., Kobayashi T., Salles J.-P., Perret B., Bonnerot C. (2004). Mast cell-and dendritic cell-derived exosomes display a specific lipid composition and an unusual membrane organization. Biochem. J..

[80] Subra C., Laulagnier K., Perret B., Record M. (2007). Exosome lipidomics unravels lipid sorting at the level of multivesicular bodies. Biochimie.

[81] Trajkovic K., Hsu C., Chiantia S., Rajendran L., Wenzel D., Wieland F., Schwille P., Brügger B., Simons M. (2008). Ceramide triggers budding of exosome vesicles into multivesicular endosomes. Science.

[82] Lunavat T.R., Cheng L., Kim D.-K., Bhadury J., Jang S.C., Lässer C., Sharples R.A., López M.D., Nilsson J., Gho Y.S. (2015). Small RNA deep sequencing discriminates subsets of extracellular vesicles released by melanoma cells–evidence of unique microRNA cargos. RNA Biol..

[83] Hunter M.P., Ismail N., Zhang X., Aguda B.D., Lee E.J., Yu L., Xiao T., Schafer J., Lee M.-L.T., Schmittgen T.D. (2008). Detection of microRNA expression in human peripheral blood microvesicles. PLoS ONE.

[84] Ji H., Chen M., Greening D.W., He W., Rai A., Zhang W., Simpson R.J. (2014). Deep sequencing of RNA from three different extracellular vesicle (EV) subtypes released from the human lim1863 colon cancer cell line uncovers distinct miRNA-enrichment signatures. PLoS ONE.

[85] Ekström K., Valadi H., Sjöstrand M., Malmhäll C., Bossios A., Eldh M., Lötvall J. (2012). Characterization of mRNA and microRNA in human mast cell-derived exosomes and their transfer to other mast cells and blood CD34 progenitor cells. J. Extracell. Vesicles.

[86] Hong B.S., Cho J.-H., Kim H., Choi E.-J., Rho S., Kim J., Kim J.H., Choi D.-S., Kim Y.-K., Hwang D. (2009). Colorectal cancer cell-derived microvesicles are enriched in cell cycle-related mRNAs that promote proliferation of endothelial cells. BMC Genom..

[87] Noerholm M., Balaj L., Limperg T., Salehi A., Zhu L.D., Hochberg F.H., Breakefield X.O., Carter B.S., Skog J. (2012). RNA expression patterns in serum microvesicles from patients with glioblastoma multiforme and controls. BMC Cancer.

[88] Palanisamy V., Sharma S., Deshpande A., Zhou H., Gimzewski J., Wong D.T. (2010). Nanostructural and transcriptomic analyses of human saliva derived exosomes. PLoS ONE.

[89] Rauschenberger L., Staar D., Thom K., Scharf C., Venz S., Homuth G., Schlüter R., Brandenburg L.O., Ziegler P., Zimmermann U. (2016). Exosomal particles secreted by prostate cancer cells are potent mRNA and protein vehicles for the interference of tumor and tumor environment. Prostate.

[90] Yang J., Wei F., Schafer C., Wong D.T. (2014). Detection of tumor cell-specific mRNA and protein in exosome-like microvesicles from blood and saliva. PLoS ONE.

[91] Taylor D.D., Gercel-Taylor C. (2008). MicroRNA signatures of tumor-derived exosomes as diagnostic biomarkers of ovarian cancer. Gynecol. Oncol..

[92] Liang X., Zhang L., Wang S., Han Q., Zhao R.C. (2016). Exosomes secreted by mesenchymal stem cells promote endothelial cell angiogenesis by transferring miR-125a. J. Cell Sci..

[93] Miller I.V., Raposo G., Welsch U., Prazeres da Costa O., Thiel U., Lebar M., Maurer M., Bender H.U., Luettichau I., Richter G.H. (2013). First identification of ewing’s sarcoma-derived extracellular vesicles and exploration of their biological and potential diagnostic implications. Biol. Cell.

[94] Shao H., Chung J., Lee K., Balaj L., Min C., Carter B.S., Hochberg F.H., Breakefield X.O., Lee H., Weissleder R. (2015). Chip-based analysis of exosomal mRNA mediating drug resistance in glioblastoma. Nat. Commun..

[95] Herrera M., Fonsato V., Gatti S., Deregibus M., Sordi A., Cantarella D., Calogero R., Bussolati B., Tetta C., Camussi G. (2010). Human liver stem cell-derived microvesicles accelerate hepatic regeneration in hepatectomized rats. J. Cell. Mol. Med..

[96] Deregibus M.C., Cantaluppi V., Calogero R., Iacono M.L., Tetta C., Biancone L., Bruno S., Bussolati B., Camussi G. (2007). Endothelial progenitor cell–derived microvesicles activate an angiogenic program in endothelial cells by a horizontal transfer of mRNA. Blood.

[97] Singer A.J., Clark R.A. (1999). Cutaneous wound healing. N. Engl. J. Med..

[98] Biró É., Sturk-Maquelin K.N., Vogel G.M., Meuleman D.G., Smit M.J., Hack C.E., Sturk A., Nieuwland R. (2003). Human cell-derived microparticles promote thrombus formation in vivo in a tissue factor-dependent manner. J. Thromb. Haemost..

[99] Berckmans R.J., Sturk A., van Tienen L.M., Schaap M.C., Nieuwland R. (2011). Cell-derived vesicles exposing coagulant tissue factor in saliva. Blood.

[100] Zhang B., Wu X., Zhang X., Sun Y., Yan Y., Shi H., Zhu Y., Wu L., Pan Z., Zhu W. (2015). Human umbilical cord mesenchymal stem cell exosomes enhance angiogenesis through the WNT4/β-catenin pathway. Stem Cells Trans. Med..

[101] Du T., Ju G., Wu S., Cheng Z., Cheng J., Zou X., Zhang G., Miao S., Liu G., Zhu Y. (2014). Microvesicles derived from human Wharton’s jelly mesenchymal stem cells promote human renal cancer cell growth and aggressiveness through induction of hepatocyte growth factor. PLoS ONE.

[102] Jeong D., Jo W., Yoon J., Kim J., Gianchandani S., Gho Y.S., Park J. (2014). Nanovesicles engineered from ES cells for enhanced cell proliferation. Biomaterials.

[103] Li X., Jiang C., Zhao J. (2016). Human endothelial progenitor cells-derived exosomes accelerate cutaneous wound healing in diabetic rats by promoting endothelial function. J. Diabetes Complicat..

[104] Huang P., Bi J., Owen G.R., Chen W., Rokka A., Koivisto L., Heino J., Häkkinen L., Larjava H. (2015). Keratinocyte microvesicles regulate the expression of multiple genes in dermal fibroblasts. J. Investig. Dermatol..

[105] Hu L., Wang J., Zhou X., Xiong Z., Zhao J., Yu R., Huang F., Zhang H., Chen L. (2016). Exosomes derived from human adipose mensenchymal stem cells accelerates cutaneous wound healing via optimizing the characteristics of fibroblasts. Sci. Rep..

[106] Zhang J., Chen C., Hu B., Niu X., Liu X., Zhang G., Zhang C., Li Q., Wang Y. (2016). Exosomes derived from human endothelial progenitor cells accelerate cutaneous wound healing by promoting angiogenesis through Erk1/2 signaling. Int. J. Biol. Sci..

[107] Guo S.-C., Tao S.-C., Yin W.-J., Qi X., Yuan T., Zhang C.-Q. (2017). Exosomes derived from platelet-rich plasma promote the re-epithelization of chronic cutaneous wounds via activation of YAP in a diabetic rat model. Theranostics.

[108] Leoni G., Neumann P.-A., Kamaly N., Quiros M., Nishio H., Jones H.R., Sumagin R., Hilgarth R.S., Alam A., Fredman G. (2015). Annexin A1-containing extracellular vesicles and polymeric nanoparticles promote epithelial wound repair. J. Clin. Investig..

[109] Bhatwadekar A.D., Glenn J.V., Curtis T.M., Grant M.B., Stitt A.W., Gardiner T.A. (2009). Retinal endothelial cell apoptosis stimulates recruitment of endothelial progenitor cells. Investig. Ophthalmol. Vis. Sci..

[110] Cheng C.-F., Fan J., Fedesco M., Guan S., Li Y., Bandyopadhyay B., Bright A.M., Yerushalmi D., Liang M., Chen M. (2008). Transforming growth factor α (TGFα)-stimulated secretion of HSP90α: Using the receptor LRP-1/CD91 to promote human skin cell migration against a TGFβ-rich environment during wound healing. Mol. Cell. Biol..

[111] Ngora H., Galli U.M., Miyazaki K., Zöller M. (2012). Membrane-bound and exosomal metastasis-associated C4. 4A promotes migration by associating with the α6β4 integrin and MT1-MMP. Neoplasia.

[112] Zhang J., Guan J., Niu X., Hu G., Guo S., Li Q., Xie Z., Zhang C., Wang Y. (2015). Exosomes released from human induced pluripotent stem cells-derived MSCs facilitate cutaneous wound healing by promoting collagen synthesis and angiogenesis. J. Transl. Med..

[113] Van Koppen A., Joles J.A., van Balkom B.W., Lim S.K., de Kleijn D., Giles R.H., Verhaar M.C. (2012). Human embryonic mesenchymal stem cell-derived conditioned medium rescues kidney function in rats with established chronic kidney disease. PLoS ONE.

[114] Gospodarowicz D. (1991). Biological activities of fibroblast growth factors. Ann. N. Y. Acad. Sci..

[115] Gastpar R., Gehrmann M., Bausero M.A., Asea A., Gross C., Schroeder J.A., Multhoff G. (2005). Heat shock protein 70 surface-positive tumor exosomes stimulate migratory and cytolytic activity of natural killer cells. Cancer Res..

[116] Atalay M., Oksala N., Lappalainen J., Laaksonen D.E., Sen C.K., Roy S. (2009). Heat shock proteins in diabetes and wound healing. Curr. Protein Pept. Sci..

[117] McCready J., Sims J.D., Chan D., Jay D.G. (2010). Secretion of extracellular HSP90α via exosomes increases cancer cell motility: A role for plasminogen activation. BMC Cancer.

[118] Pearl L.H., Prodromou C. (2000). Structure and in vivo function of HSP90. Curr. Opin. Struct. Biol..

[119] Liekens S., de Clercq E., Neyts J. (2001). Angiogenesis: Regulators and clinical applications. Biochem. Pharmacol..

[120] Hughes C.C. (2008). Endothelial–stromal interactions in angiogenesis. Curr. Opin. Hematol..

[121] Yuan H., Guan J., Zhang J., Zhang R., Li M. (2016). Exosomes secreted by human urine-derived stem cells accelerate skin wound healing by promoting angiogenesis in rat. Cell Biol. Int..

[122] Li X., Chen C., Wei L., Li Q., Niu X., Xu Y., Wang Y., Zhao J. (2016). Exosomes derived from endothelial progenitor cells attenuate vascular repair and accelerate reendothelialization by enhancing endothelial function. Cytotherapy.

[123] Vrijsen K.R., Maring J.A., Chamuleau S.A., Verhage V., Mol E.A., Deddens J.C., Metz C.H., Lodder K., van Eeuwijk E., van Dommelen S.M. (2016). Exosomes from cardiomyocyte progenitor cells and mesenchymal stem cells stimulate angiogenesis via emmprin. Adv. Healthc. Mater..

[124] Ramakrishnan D.P., Hajj-Ali R.A., Chen Y., Silverstein R.L. (2016). Extracellular vesicles activate a CD36-dependent signaling pathway to inhibit microvascular endothelial cell migration and tube formationsignificance. Arterioscler. Thromb. Vasc. Biol..

[125] Nakamura K., Jinnin M., Harada M., Kudo H., Nakayama W., Inoue K., Ogata A., Kajihara I., Fukushima S., Ihn H. (2016). Altered expression of CD63 and exosomes in scleroderma dermal fibroblasts. J. Dermatol. Sci..

[126] Jong O.G., Balkom B.W., Gremmels H., Verhaar M.C. (2016). Exosomes from hypoxic endothelial cells have increased collagen crosslinking activity through up-regulation of lysyl oxidase-like 2. J. Cell. Mol. Med..

[127] Huleihel L., Hussey G.S., Naranjo J.D., Zhang L., Dziki J.L., Turner N.J., Stolz D.B., Badylak S.F. (2016). Matrix-bound nanovesicles within ECM bioscaffolds. Sci. Adv..

[128] Wilhelm E.N., González-Alonso J., Parris C., Rakobowchuk M. (2016). Exercise intensity modulates the appearance of circulating microvesicles with proangiogenic potential upon endothelial cells. Am. J. Physiol. Heart Circ. Physiol..

[129] Atienzar-Aroca S., Flores-Bellver M., Serrano-Heras G., Martinez-Gil N., Barcia J.M., Aparicio S., Perez-Cremades D., Garcia-Verdugo J.M., Diaz-Llopis M., Romero F.J. (2016). Oxidative stress in retinal pigment epithelium cells increases exosome secretion and promotes angiogenesis in endothelial cells. J. Cell Mol. Med..

